# The Effect of JAK1/2 Inhibitors on HIV Reservoir Using Primary Lymphoid Cell Model of HIV Latency

**DOI:** 10.3389/fimmu.2021.720697

**Published:** 2021-08-31

**Authors:** Lesley R. de Armas, Christina Gavegnano, Suresh Pallikkuth, Stefano Rinaldi, Li Pan, Emilie Battivelli, Eric Verdin, Ramzi T. Younis, Rajendra Pahwa, Siôn L. Williams, Raymond F. Schinazi, Savita Pahwa

**Affiliations:** ^1^Department of Microbiology and Immunology, University of Miami Miller School of Medicine, Miami, FL, United States; ^2^Department of Pathology and Experimental Medicine, Emory University and Children’s Healthcare of Atlanta, Atlanta, GA, United States; ^3^Department of Pharmacology and Chemical Biology, Emory University and Children’s Healthcare of Atlanta, Atlanta, GA, United States; ^4^Center for AIDS Research, Department of Pediatrics, Emory University and Children’s Healthcare of Atlanta, Atlanta, GA, United States; ^5^Gladstone Institute of Virology and Immunology, Gladstone Institutes, San Francisco, CA, United States; ^6^Department of Medicine, University of California San Francisco, San Francisco, CA, United States; ^7^Buck Institute for Research on Aging, Novato, CA, United States; ^8^Department of Otolaryngology, University of Miami Miller School of Medicine, Miami, FL, United States; ^9^Department of Neurology, University of Miami Miller School of Medicine, Miami, FL, United States

**Keywords:** HIV, latency, tonsil, scRNAseq, JAK-STAT signaling pathway, LRA (latency reversing agent)

## Abstract

HIV eradication is hindered by the existence of latent HIV reservoirs in CD4^+^ T cells. Therapeutic strategies targeting latent cells are required to achieve a functional cure, however the study of latently infected cells from HIV infected persons is extremely challenging due to the lack of biomarkers that uniquely characterize them. In this study, the dual reporter virus HIV_GKO_ was used to investigate latency establishment and maintenance in lymphoid-derived CD4^+^ T cells. Single cell technologies to evaluate protein expression, host gene expression, and HIV transcript expression were integrated to identify and analyze latently infected cells. FDA-approved, JAK1/2 inhibitors were tested in this system as a potential therapeutic strategy to target the latent reservoir. Latent and productively infected tonsillar CD4^+^ T cells displayed similar activation profiles as measured by expression of CD69, CD25, and HLADR, however latent cells showed higher CXCR5 expression 3 days post-infection. Single cell analysis revealed a small set of genes, including *HIST1*-related genes and the inflammatory cytokine, *IL32*, that were upregulated in latent compared to uninfected and productively infected cells suggesting a role for these molecular pathways in persistent HIV infection. *In vitro* treatment of HIV-infected CD4^+^ T cells with physiological concentrations of JAK1/2 inhibitors, ruxolitinib and baricitinib, used in clinical settings to target inflammation, reduced latent and productive infection events when added 24 hr after infection and blocked HIV reactivation from latent cells. Our methods using an established model of HIV latency and lymphoid-derived cells shed light on the biology of latency in a crucial anatomical site for HIV persistence and provides key insights about repurposing baricitinib or ruxolitinib to target the HIV reservoir.

## Introduction

The latent HIV reservoir is defined as cells carrying integrated, replication-competent provirus that do not express viral transcripts or proteins (1). CD4^+^ T cells are recognized as the predominant cellular refuge for the latent HIV reservoir; specifically resting memory cells have been shown to be enriched in HIV provirus compared to other CD4^+^ T cell subsets, however cells from the myeloid lineage can also be infected by HIV and contribute to persistence of latency (2–4). Studies of antiretroviral therapy (ART) initiation during acute infection in humans and non-human primates (NHP) have shown that the latent reservoir is established very early following HIV infection (5, 6). This is likely due to the ability of latently infected cells to distribute throughout the tissues with a higher viral burden detected in the gut, lymph nodes, and CNS relative to peripheral blood (7–9).

The persistence of latent HIV reservoirs despite effective ART remains the largest barrier to the cure of HIV/AIDS (10–12). Many technical challenges contribute to the difficulties in studying latent HIV infection. First, in patients under ART suppression latently infected cells are present at very low frequencies in the blood with estimates at 1-1,000 per million CD4^+^ T cells (13, 14). Second, despite this being an area of intense research that has generated several candidate markers including CD32a, CD30, PD-1, Lag-3, Tigit, and CD127 (15–21), there are no validated phenotypic biomarkers to distinguish latently infected from uninfected cells. Third, the majority of latent HIV genomes detected in ART-treated subjects are defective and will not produce infectious virions upon viral reactivation (i.e., transcription) (22). Finally, the two methods generally accepted for quantifying the replication-competent HIV reservoir, quantitative viral outgrowth assay (QVOA) (22) and intact proviral DNA assay (IPDA) (13), do not allow for direct interrogation of latently-infected cells and thereby hinder the discovery of biomarkers and the study of mechanisms governing latency. These stringencies limit our ability to apply mechanistic pharmacological intervention studies, with a goal of understanding the direct impact of an agent on the replication competent reservoir, which is sentinel towards informed translational research that can be applied towards human studies in the cure space.

To overcome the hurdle of trying to study extremely rare latently infected CD4^+^ T cells *in vivo*, *in vitro* models have been generated (23–25). Primary CD4^+^ T cell models are especially useful and easily established using cells from HIV negative donors. A widely used model for HIV latency involves selection of resting CD4^+^ T cells (negative for expression of T cell activation markers CD69, HLADR, CD25) that have been stimulated with CCR7 ligands to support viral integration with limited viral replication (24, 26–28). Primary cell models use *in vitro* infection with HIV viruses of varying subtypes (e.g. B or C) and envelope tropism (29) and assess infection efficiency through the use of Gag p24 detection or fluorescent reporter expression indicating productive infection. Unfortunately, latently infected cells are still undetectable using these techniques and the approach given this limitation is to allow confirmed infected cells (e.g., GFP^+^) ample time in culture (2-8 weeks) to silence HIV transcription and convert from productive to latent (25, 30).

On the other hand, dual reporter viral constructs allow for direct and simultaneous detection of HIV infected cells at different stages (i.e., latent and productive). HIV_GKO_ is a second-generation dual reporter virus and features eGFP marker under the control of the HIV LTR promoter and a Kusabira Orange 2 (mKO2) fluorescent marker under the control of the host elongation factor 1a (EF1α) promoter (31–35). Given the propensity for HIV infected cells to be recovered from lymphoid tissues (7, 36–38), we used HIV_GKO_ to investigate latency establishment and maintenance in lymphoid-derived, tonsillar CD4^+^ T cells. Using this system, we were able to integrate datasets from single cell technologies to evaluate protein expression, host gene expression, and HIV transcript expression to characterize latently infected cells.

Finally, we used this tool to test reactivation of latency and FDA-approved JAK1/2 inhibitors as a therapeutic intervention for silencing HIV transcription. The FDA-approved JAK1/2 inhibitor ruxolitinib was recently evaluated in an AIDS Clinical Trial Group multi-site Phase 2a study (A5336), and demonstrated safety and efficacy in virally suppressed people living with HIV, including a significant decrease in key markers associated with HIV persistence including HLA-DR/CD38, CD25, and sCD14, as well as cellular/reservoir lifespan marker Bcl-2 (39). Baricitinib is a second-generation orally bioavailable JAK1/2 inhibitor that has an improved safety profile *versus* ruxolitinib, is approved for chronic long-term use in adults and children as young as two years of age (Olumiant.com). *In vitro*, ruxolitinib treatment of HIV-infected CD4^+^ T cells inhibits virus production, STAT5 phosphorylation, homeostatic proliferation, and Bcl-2 downregulation (40). These findings prompted us to explore the ability of the second-generation JAK1/2 inhibitor baricitinib to target the latent reservoir directly.

## Methods

### Specimen Collection

Tonsil samples were obtained from HIV-negative children and adolescents during elective tonsillectomy for sleep apnea at University of Miami Hospitals with informed consent. Single cell suspensions of mononuclear cells were isolated from tonsil tissue by mechanical separation and then filtered through a 70-micron filter in RPMI 1640 (Gibco) as described previously (41). Mononuclear cells were cryopreserved in FBS containing 20% DMSO and stored in liquid nitrogen freezers.

### Virus Production

Plasmid DNA for the dual reporter viral construct, HIV_GKO_, was obtained from Eric Verdin and Emilie Battivelli (Gladstone Institute, UCSF) and the dual tropic envelope construct was obtained through the NIH HIV Reagent Program, Division of AIDS, NIAID, NIH: Plasmid pSVIII Expressing HIV-1 92HT593.1 gp160, ARP-3077, contributed by Dr. Beatrice Hahn. Plasmids were transformed in chemically competent E. coli and plasmid DNA was expanded and isolated using Maxi-Prep (Qiagen) reagent according to manufacturer’s instructions. 293FT cell lines (Invitrogen) were used to produce virus particles and were cultured in DMEM (containing 4,500 mg/L D-glucose no L-glutamine or sodium pyruvate) supplemented with 10% (v/v) FBS, 4 mM of L-glutamine, 110 mg/L (1mM) sodium pyruvate and 1% penicillin/streptomycin. Cells were transfected with both plasmids when culture flasks showed 90-95% confluency. 24 hours before transfection the culture media was changed for antibiotic-free media. Briefly, plasmid DNA was diluted in Opti-Mem media (Invitrogen) at a ratio of 1.8:1 (HIV_GKO_: pSVIII) before mixing with Lipofectamine 2000 Transfection Reagent (Invitrogen) according to the manufacturer’s instructions. The day after transfection (32-36 hr post-transfection) the supernatant was removed and replaced with fresh antibiotic-free media. The following morning, viral supernatants were harvested into 50ml tubes and centrifuged at 1000*g* for 4 min to remove cellular debris. The supernatants were filtered through a 0.45µM low protein binding filter and loaded into ultracentrifuge tubes. Virus was concentrated by ultracentrifugation (27,000*g* at 4C for 2.3 hr), resuspended by trituration in antibiotic-free DMEM medium, aliquoted, and stored at -80°C. Viral titers were quantified using p24 ELISA kits (Perkin Elmer).

### *In Vitro* Infection With HIV_GKO_

Cryopreserved tonsil mononuclear cells were thawed and cultured in complete medium RPMI (Invitrogen) supplemented with 10% FBS, L-glutamine, and Penicillin/Streptomycin overnight. CD4^+^ T cells were purified using EasySep™ Human CD4^+^ T cell enrichment kit (StemCell Technologies) by negative selection. Purified CD4^+^ T cells were cultured at a concentration of 2-5 million/mL of complete medium and activated using soluble anti-CD3 (1 µg/mL) and anti-CD28 (1ug/ml) antibodies for 3 days in a 37°C, 5%CO_2_ incubator. Activated CD4^+^ T cells were infected with HIV_GKO_ (100 ng p24/million cells) by spinoculation at 1,200*g* for 2.2 hr. Cell pellets were resuspended in complete medium containing IL-2 (30 U/mL, Peprotech) and cultured until further analysis.

### Flow Cytometry Acquisition and Sorting

**Figure 1:**
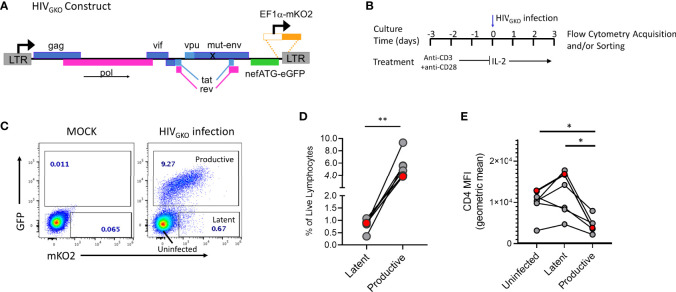
HIV_GKO_ infection in tonsil derived CD4^+^ T cells. **(A)** HIV_GKO_ viral construct showing mutated *env* gene, *nef* gene replaced by an eGFP reporter, and insertion of EF1a promoter directly upstream of mKO2 reporter. **(B)** Schematic showing experimental design for activation and infection with HIV_GKO_ of purified tonsillar CD4^+^ T cells. **(C)** Representative flow plot showing expression of dual fluorescent reporters from HIV_GKO_ on day 3 post-infection. **(D)** Summary data from 6 tonsil donors with frequency of productive and latent infected cells based on gating shown in panel **(C)**. **(E)** Mean fluorescent intensity of cell surface protein expression for CD4 on cells infected with HIV_GKO_ on day 3 post-infection. Red data points in panels D and E indicate the individual donor used for single cell analyses in single cell RNA Seq experiments. Paired t test was performed to compare Uninfected, Latent, and Productive cell populations, *p < 0.05, **p < 0.01.

For phenotypic analysis of HIV_GKO_ infected cells by flow cytometry, cells were labeled using fluorochrome-conjugated monoclonal antibodies against CD4, CD45RO, CD69, HLADR, CD25, PD-1, and CXCR5 as previously described (42) (see **Supplemental Table 1** for Ab details). Cells were also labeled with violet live/dead stain (VIVID, Molecular Probes) for dead cell exclusion and acquired on LSRII (Becton Dickinson) or SH800 Sorter (Sony). Flow cytometry data was analyzed using FlowJo (Version 10.7.1, TreeStar). Gating for productive and latent cell populations were based off of mock-infected sample (as shown in **Figure 1C**). Gating for cell surface markers were determined using unstained control samples to define negative gates. At the time of panel validation, FMO (fluorescence minus one) controls were used to define compensation parameters and compatibility of the different markers. Additionally, all antibodies were titrated for optimal concentration for labeling.

### Single Cell 3’ Whole Transcriptome Amplification by BD Precise Assay

Single cell sorting was performed using the Sony SH800 instrument and ‘single cell’ sorting mode and 100uM chip. Individual cells were sorted into each well of BD Precise Assay 96 well plates based on gating for productive, latent, or uninfected cells as shown in **Figure 1A**. Immediately after sorting the plate was centrifuged at 1,000*g* for 5 min and stored at -80°C until library preparation was performed. Next-generation sequencing (NGS) library preparation was performed according to manufacturer’s instructions for BD Precise Assays for patented BD™ Molecular Indexing (MI) technology with Sample Index (SI) to label individual mRNA transcripts. Briefly, during reverse transcription, the BD Precise assay applies a non-depleting pool of 65,536 barcodes termed molecular indexes (MI) for stochastic and unique labeling of mRNA transcripts. In addition to MI, a second set of barcodes (sample index, SI) are employed to identify the sample origin of each transcript according to the well position in the 96-well plate. Barcoded primers are then used to label polyadenylated RNA transcripts in each of the 96 wells, followed by a pooling step into a single tube. The resulting library was then input onto Illumina MiSeq sequencer using the appropriate sequencing kits. Each of the sequencing reads were processed to identify the MI, SI and target gene using BD primary analysis pipeline. To mitigate the effect of over-estimation of molecules from PCR and sequencing errors, the BD analysis pipeline contains Molecular Identifier (MI) adjustment algorithms recursive substitution error correction (RSEC) and distribution-based error correction (DBEC). A gene is subjected to DBEC if it meets a certain threshold for sequencing depth. If a gene passes the threshold for DBEC, the status is pass. If a gene does not pass, the status is low depth. If a gene has zero counts across all cells, the status is not detected. The MI counts detected in each plate showed comparable and similar distribution of Pass and Low Depth (see *Methods*) transcripts (**Supplemental Figure 1**). scRNA Seq data was also analyzed based on fluorescent classifications of infection status and showed comparable MI counts. Blank wells generated at the time of sorting had low MI counts compared to cell-containing wells recognizing a couple hundred genes (compared to ~17,000 for cell-containing wells) across 15 blank wells but with very low read counts per gene ≤ 3.

### RT-PCR for IL32 Expression

Bulk sorting of productive, latent, and uninfected cells were sorted according to gating shown in **Figure 1C**. Cells (500) were sorted directly into CellsDirect one-step qRT-PCR reagents (Invitrogen) including primers for IL32 and GAPDH (Taqman Assay 1:100, ABI), 2x CellsDirect reaction mix, water, and SuperScript^®^ III Reverse Transcriptase and Platinum^®^ Taq DNA Polymerase. Eighteen cycles of pre-amplification were performed as previously described (43). The resulting cDNA was analyzed in standard Taqman qRT-PCR to assess gene transcript levels for IL32 and GAPDH. Relative quantification was performed by calculating ddCT values.

### Cell Sorting and HIV Reactivation Assay

Purified CD4^+^ T cells were infected with HIV_GKO_ as described above. On day 5 post-infection, cells were labeled with live/dead (VIVID) stain and GFP negative, latent cell-enriched gated population was sorted in purity mode using Sony SH800 sorter. The sorting gate contained ~10% of latent cells by fluorescent reporter expression (mKO2+GFP-) and the rest were uninfected (GFP-). Cells were collected in complete medium (R10), centrifuged, and resuspended in fresh medium and divided equally into 4 wells of a 96 well plate with approximately 10^4^ cells/well in 150ul of R10. The cells rested overnight in presence or absence of physiologically relevant concentrations of (0.1, 1, and 10 µM) baricitinib (Selleck chemicals) before addition of LRA (1 µg/mL each of anti-CD3 and anti-CD28). Two days after LRA treatment, cells were stained for live/dead (VIVID) and acquired on Sony SH800 sorter to assess GFP and mKO2 expression for at least 25,000 cells (events). Flow cytometric data were analyzed using FlowJo version 10.7.1.

### Statistical Analysis

Single cell gene expression analysis was performed using R fluidigmSC package from SINGuLAR™ Analysis Toolset which is designed specifically for single-cell studies of gene expression profiles. Pathway analysis was performed using QIAGEN Ingenuity Pathway Analysis, foldchange cutoff is 1.5, p value cutoff 0.05.

### Study Approval

This study was approved by the Institutional Review Boards of University of Miami and was conducted in accordance with approved guidelines (IRB# 20140200/CR00004416). Voluntary signed informed consent was obtained from every participant prior to participating in the study.

## Results

### Latency Is Established Early Following HIV_GKO_ Infection of Tonsil-Derived CD4^+^ T Cells

To identify and study latently infected lymphoid-derived cells, we performed *in vitro* infection of tonsillar CD4^+^ T cells from HIV negative donors using the lentiviral vector HIV_GKO_ (**Figure 1A**), pseudo-typed with an X4/R5 dual tropic HIV-1 envelope subtype B. In this model, purified CD4^+^ T cells are TCR-activated prior to infection with HIV_GKO_ and evaluated 3 days after infection by flow cytometry to assess frequencies of productive and latent infected cells (**Figure 1B**). GFP and mKO2 expression were used to distinguish between the different populations compared to a mock-infected control (**Figure 1C**). mKO2 expression driven by the host-derived EF1α promoter activity in the cell nucleus signals the presence of an integrated viral genome in the cell, thus mKO2 expression in the absence of GFP expression defines the latently infected cell population in this system. GFP-expressing cells were considered productively infected though expression of mKO2 was variable, due to fluctuating expression of the EF1α promoter (44). HIV_GKO_ infection consistently established latent infection (GFP-mKO2+) in purified CD4^+^ T cells from different tonsil donors (**Figure 1D**). However, latent infection was always established at lower frequencies compared to productive infection (GFP+) (mean 0.83% *vs* 5.5%, respectively). Productive cells infected with HIV_GKO_ also exhibited reduced CD4 expression compared to latent and uninfected cells (**Figure 1E**) (mean CD4 MFI: productive 4,533 *vs* 11,747 and 9,633 for latent and uninfected, respectively).

### CD4^+^ T Cells With Latent Infection Can Exhibit Similar Activation Profiles as Productive Infected Cells

**Figure 2:**
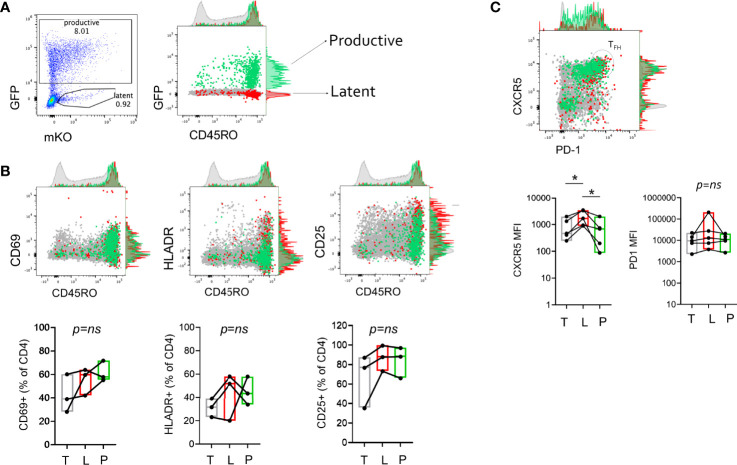
Cellular activation and differentiation marker expression on productive and latent infected CD4^+^ T cells. **(A)** Measurement of memory marker CD45RO expression on productive (P, GFP+, green), Latent (L, GFP-mKO+, red), and total (T, light gray) CD4^+^ T cells 3 days post-infection with HIV_GKO_. **(B)** Representative plots showing expression of activation markers (CD69, HLADR, CD25) and CD45RO expression on productive, latent, and total cells. **(C)** Representative plot showing expression of TFH markers, CXCR5 and PD-1 on productive, latent, and total cells. CXCR5^hi^PD-1^hi^ cells define TFH population. Box and whisker plots show data from 3-5 independent experiments (individual tonsil donors). Paired t-test analysis was performed to determine differences between the groups, *p < 0.05, ns, not significant.

To characterize the cells harboring latent virus in lymphoid CD4^+^ T cells generated in the HIV_GKO_ model, surface expression of memory and activation markers 3 days after infection were assessed by flow cytometry. Productive, Latent, and uninfected cells were assessed for expression of CD45RO, CD69, HLADR, and CD25. Infected cells were enriched in the CD45RO^+^ fraction compared to total cells, though a small fraction of infected cells were CD45RO negative (**Figure 2A** and **Supplemental Table 2**). To test the hypothesis that latent infection was the result of a transition from activated to resting state (23, 33, 45) we reasoned that productive-infected cells should have higher expression of activation markers compared to latent. However, expression of activation markers was variable on infected cells with no apparent enrichment or preference for expression of a particular marker on productive *vs.* latent cells (**Figure 2B**). T follicular helper cells (Tfh) have been shown to harbor replication competent virus in lymph nodes (8, 46), thus we assessed markers for Tfh phenotype, CXCR5 and PD-1 to determine if there was any preference for latency establishment within this subset. There was donor variation in the infection efficiency within the Tfh population (CXCR5^hi^PD-1^hi^), however evaluation of MFI of each marker individually showed that latent infected cells had higher CXCR5 MFI compared to productive and uninfected (p < 0.05) (**Figure 2C**). This association was not observed for PD-1.

### Using Single-Cell RNASeq to Discover Potential Biomarkers of Latency

An important advantage of the HIV_GKO_ is that using FACS sorting, mKO2^+^ cells can be isolated without the need for virus reactivation providing a rich source of unperturbed latent cells to study gene expression and identify potential biomarkers of latency. For single cell gene expression studies, we selected one tonsil donor for downstream single cell RNA Seq analysis (donor highlighted in red datapoints in **Figures 1D, E**) in an effort to eliminate donor variation in the subsequent gene expression data. To generate RNA Seq data, we employed a plate-based platform for scRNA Seq (BD™ Precise assay) to the HIV_GKO_ latency model and performed whole transcriptome amplification (WTA) from a total of 462 cells from five 96-well plates across three independent experiments using a single tonsil donor. Each plate contained equal numbers of productive, latent, and uninfected cells as determined by gating using expression of the fluorescent reporters, GFP and mKO2 (index sorting data shown in **Supplemental Figure 2**). GFP^+^ cells had varying expression of mKO2, suggesting differential activity of the EF1α promoter in this model, therefore, to exclude effects related to EF1α activity only productive cells with mKO2 expression matching that of latent cells were sorted.

We first evaluated specific genes related to T cell activation and T helper subset differentiation within each of the sorted populations (**Supplemental Figure 3**). Transcripts for CD69 and HLADR proteins had higher expression in productive cells and expression decreased with the level of HIV transcript expression (i.e. productive>latent>uninfected). Genes related to cytokine and transcription factor expression for Th1 (*TBX21, IFNG*) and Th2 (*GATA3, IL13*) type cells did not show enrichment for either sorted population, however the Th17 transcription factor *RORC* was increased in productive compared to uninfected cells with a trend toward increased expression with greater HIV transcript expression.

**Figure 3:**
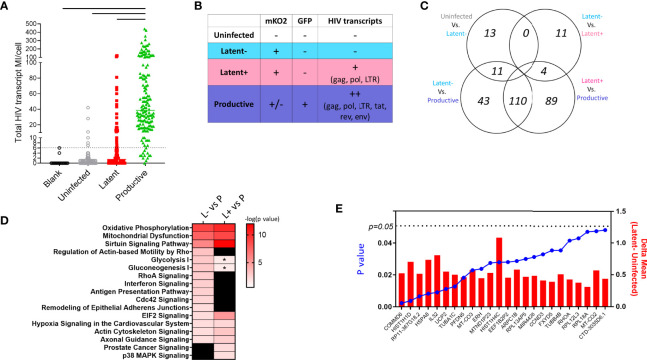
Single cell RNA Seq in HIV_GKO_ model of latency. **(A)** HIV transcript MI counts per cell in each sorted cell type. Dotted line is cutoff for background levels of transcript expression based on blank control wells in which no cells were sorted. Solid lines indicate significant difference between productive cells and all other groups using ANOVA one-way test with multiple comparisons (p < 0.05). **(B)** Schematic showing cell characterization based on fluorescent reporter and HIV transcript expression. **(C)** Venn diagram showing number of differentially expressed genes between the cell populations defined in **(B)**. **(D)** Heatmap showing shared enriched pathways between Latent cell populations and productive cells. Colored boxes indicate significant pathways p < 0.01, * indicates pathways with p < 0.05 and > 0.01. **(E)** Bar graph showing the difference in average expression of each DEG between Latent and Uninfected cells with p values from ANOVA analysis shown as a super-imposed line graph (blue).

To confirm the presence (or absence) of HIV transcription in the HIV_GKO_ infected cells, we aligned scRNA Seq data against the HIV_GKO_ sequence to assess expression of open reading frames (ORF) for gag, pol, tat, rev, vpr, and env genes in sorted single cells (**Figure 3A**). Productive cells expressed highest HIV transcripts per cell (median 38.5) as expected by high levels of LTR-driven GFP reporter expression (**Figure 3A**). Latent infected cells had a median of 1.5 HIV transcripts per cell, however, we observed a fraction of cells with HIV transcript levels matching GFP^+^ population (22.7% with >6 MI/cell). Overall, the majority of uninfected and latent cells exhibited HIV transcript levels below the cutoff established by analysis of blank wells (no sorted cell). Desiring to have pure populations of uninfected, latent, and productive cells for gene expression analysis, we re-classified and grouped single cells on the basis of fluorescent reporter expression and HIV transcript levels (**Figure 3B**).

We performed differential gene expression analysis between 1) Uninfected HIV transcript negative (U-neg) and Latent HIV transcript negative (L-neg), 2) L-neg and Latent HIV transcript low+ (L+), 3) L-neg and Productive HIV transcript++ (P++), and 4) L+ and P++ (**Figure 3C**). The number of DEGs was highest in the comparisons of latent populations (L-neg and L+) *vs* productive with 164 and 203 DEGs respectively, while 110 DEGS from each comparison were overlapping (**Supplemental Data 1**). However, pathway analysis demonstrated that despite having several unique DEGs, the pathways involved were similar when comparing latent *vs* productive (**Figure 3D** and **Supplemental Data 2**). Enriched pathways included Sirtuin Signaling, Oxidative Phosphorylation, Mitochondrial Dysfunction, and EIF2 Signaling. In line with this observation, the contrast of gene expression between L-neg and L+ resulted in only 15 DEGs, exhibiting the high degree of similarity between the two populations (**Figure 3C**).

### Gene Profiles in Latent Infected Lymphoid CD4^+^ T Cells

**Figure 4:**
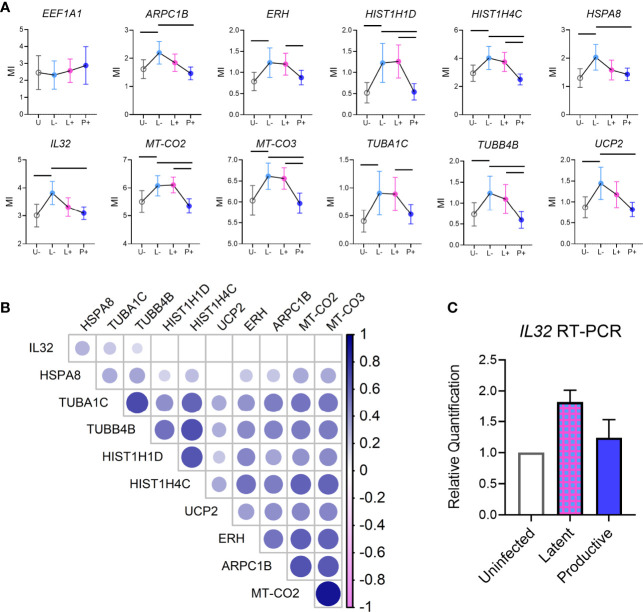
Unique gene signature in latent cells. **(A)** Individual graphs showing mean (error bars indicate 95% Confidence Interval) expression of each of the genes identified in Differentially Expressed Gene analysis between Latent cells and other infected populations (from **Figures 3B, C**). EEF1A1 expression (gray box, top left) was used as a control for EF1α promoter activity in cells. **(B)** Correlation matrix showing the co-expression of each of the genes from **(A)** on a single cell level. Color intensity is associated with the spearman correlation coefficient and the size of circle is related to the p value (larger circle, smaller p value). **(C)** Validation of IL32 expression in bulk sort-purified cells from 2 donors by RT-PCR.

We were specifically interested in the latent-infected cells and identifying a gene signature that could discriminate them from uninfected cells. To this end, we identified 24 genes with differential expression in latent *vs* uninfected cells, all of which were upregulated in L-neg (**Figure 3E**). The genes were involved in protein folding (HSPA8, TUBA1C, TUBB4B, PFDN5), protein targeting/viral gene expression (RPS19, RPL18A, RPL31, RPL39), and respiratory electron transport chain (UCP2, UCP3, MT-CO2, MT-CO3) (**Supplemental Figure 4**, Genemania network analysis). We interrogated this short list of DEGs to identify 11/24 genes that exhibited enriched expression in latent cells relative to both uninfected and productive populations, indicating a potential biomarker of latency (**Figure 4A**). As a control we evaluated gene expression for EF1α (EEF1A) in each of the populations and confirmed there were no significant differences in the groups. Co-expression analysis showed strong correlation coefficients between most of the genes indicating a common pathway involving histone modification and electron transport, however *HSP8* and especially *IL32* showed low co-expression with the other genes suggesting these two genes were not related to the common pathway (**Figure 4B**, **Supplemental Figure 5**). We confirmed by RT-PCR in sorted populations (uninfected, latent, productive) that *IL32* was upregulated in latent compared to uninfected cells (**Figure 4C**).

### Reactivation of Latency in HIV_GKO_ Infected Cells and Therapeutic Intervention With JAK1/2 Inhibitors

**Figure 5:**
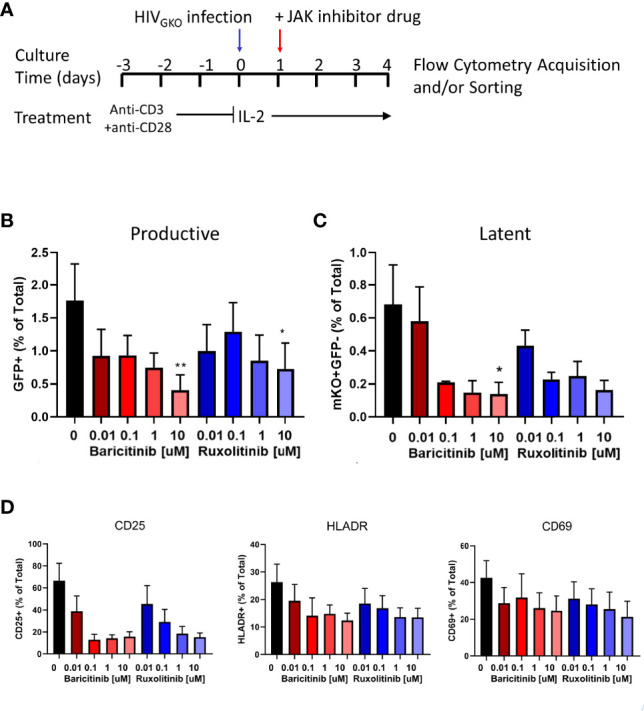
Effect of JAK1/2 inhibitors on productive and latent infection. **(A)** Schematic showing experimental design for activation and infection with HIV_GKO_ of purified tonsillar CD4^+^ T cells. Bar graphs showing average frequency of **(B)** GFP^+^ (productive) cells and **(C)** mKO+GFP- (latent) cells on day 4 post-infection in presence of JAK1/2 inhibitors at different concentrations. Error bars represent standard error of the mean (SEM), students t test was used to compare all conditions against the no drug control (black bar), *p < 0.05, **p < 0.01. **(D)** Bar graphs showing average frequency of cells expressing activation markers on day 4 post-infection in presence of JAK1/2 inhibitors at different concentrations. Results shown represent 3 independent experiments.

In addition to biomarker discovery, the major utility of a primary HIV latency model is to test therapeutic strategies targeting the latent reservoir, such as JAK1/2 inhibitors. Exogenous addition of ruxolitinib or baricitinib, to HIV_GKO_ infected cells reduced frequencies of productive (GFP^+^) and latent (GFP-mKO2^+^) infected cells in a dose-dependent manner with the strongest effects observed at the 10 µM concentration (**Figures 5A, B**). Molecular JAK1/2 inhibition also resulted in dose-dependent downregulation of activation markers on CD4^+^ T cells (**Figure 5C**). CD25 expression was reduced by >80% at the highest concentration of ruxolitinib and baricitinib, while HLADR and CD69 exhibited a reduction of 60% and 50%, respectively, with both drugs (**Figure 5D**).

**Figure 6:**
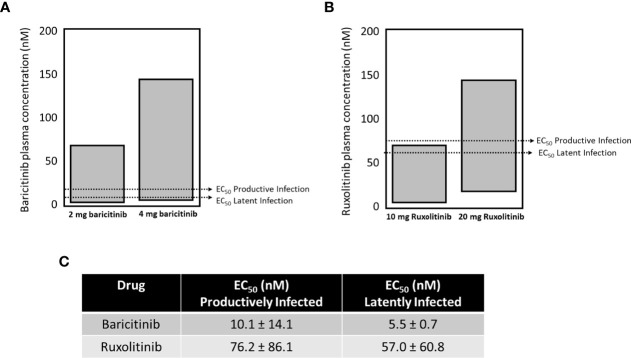
The EC_50_ for blocking seeding of productive and latent infection with baricitinib and ruxolitinib. The approved dose for chronic long-term use of baricitinib is 2 mg (USA), or 2 and 4 mg (Japan, other non-USA jurisdictions), and 4 mg is approved for hospitalized COVID-19 patients. The plasma concentrations for both doses of baricitinib fall within the range to effectively block both productive and latent infection in our single cycle model **(A)**. Ruxolitinib is not approved for chronic long-term use, but approved doses range within 10-25 mg **(B)**. Only the higher dose of ruxolitinib fell within plasma concentration ranges to block both productive and latent infection in our single cycle model **(B)**. Baricitinib demonstrates ~ half a log greater potency for both assays *versus* ruxolitinib (summarized in **C**).

The approved dose for chronic long-term use of baricitinib is 2 mg (USA), or 2 and 4 mg (Japan, other non USA jurisdictions), and 4 mg is approved for hospitalized COVID-19 patients (OLUMIANT.COM, covid19treatmentguidelines.nih.gov). The plasma concentrations for both doses of baricitinib fall within the range to effectively block both productive and latent infection in our single round replication HIV_GKO_ model (**Figure 6A**). Ruxolitinib is not approved for chronic long-term use, but approved doses range within 10-25 mg (**Figure 6B**). Only the higher dose of ruxolitinib fell within plasma concentration ranges to block both productive and latent infection. Baricitinib demonstrates ~ half a log greater potency for both assays *versus* ruxolitinib (summarized in **Figure 6C**).

**Figure 7:**
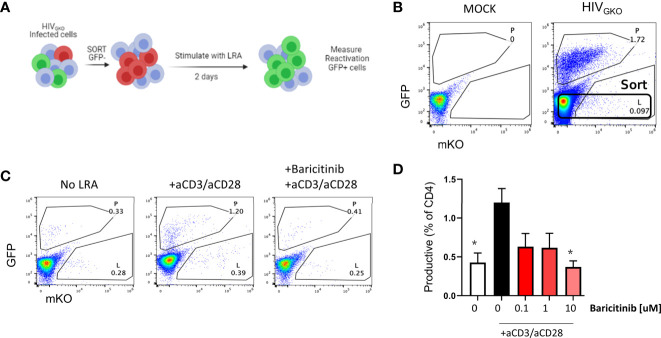
Baricitinib inhibits viral reactivation in latent HIV_GKO_ infected CD4^+^ T cells. **(A)** Schematic showing experimental design for sorting and re-activation of HIV_GKO_ in purified tonsillar CD4^+^ T cells. **(B)** Representative flow plot showing expression of dual fluorescent reporters from HIV_GKO_ on day 3 post-infection and the sorting gate for reactivation experiments. **(C)** Dot plots showing expression of dual fluorescent reporters from HIV_GKO_ on day 2 following *in vitro* reactivation. **(D)** Summary bar graph showing average frequency of GFP^+^ (productive) cells on day 2 post-LRA treatment in presence of Baricitinib at different concentrations. Error bars represent standard error of the mean (SEM), students t test was used to compare all conditions against the no drug control (white bar), *p < 0.05. Results shown represent 3 independent experiments.

Finally, we designed an experiment to evaluate reactivation from latency using HIV_GKO_ infected cells as shown in **Figure 7A**. At day 3 post infection, GFP negative cells were sorted (**Figure 7B**) and cultured with or without baricitinib at different concentrations for 24 hours prior to TCR stimulation as a latency reversing agent (LRA). In the absence of drug and TCR stimulus *via* anti-CD3/anti-CD28, spontaneous reactivation was observed indicated by the presence of GFP^+^ cells. HIV reactivation above the level of spontaneous reactivation was observed in the presence of LRA, however pretreatment of cells with baricitinib abrogated the response with the greatest effect occurring at the highest concentration of drug (**Figures 7C, D** **)**.

## Discussion

Primary models of HIV latency are important to enable research into mechanisms of HIV establishment and maintenance. The use of the dual reporter virus HIV_GKO_ in our study and others (31, 34, 35, 47) have validated that this model recapitulates multiple aspects of latent HIV infection. The decision to infect primary CD4^+^ T cells from tonsils rather than blood was made in order to shed light on latency establishment and maintenance in lymphoid derived cells which are critical for HIV reservoir establishment and persistence (9, 46, 48, 49). T follicular helper (Tfh) cells within lymph nodes and their counterpart in the blood peripheral Tfh (pTfh) represent a preferred cellular site of HIV reservoir (8, 50). Tfh harboring latent HIV may achieve protection from CTL recognition by hiding in follicles where CD8+ T cells either cannot enter due to low levels of the chemokine receptor, CXCR5 expression or are not effective at killing virus-infected cells (51, 52). Our results showed that early in the establishment of latency, tonsil CD4^+^ T cells upregulated surface expression of CXCR5 which would allow them to enter the GC follicle. These results support a mechanism of lymph node homing as a means for HIV reservoir persistence.

An advantage of the HIV_GKO_ dual reporter system is the ability to assess uninfected and infected cells from the same treatment conditions, thereby reducing ‘noise’ in the data. Primary cell HIV latency models tend to be long-term experiments taking 2-12 weeks to obtain a pool of latently infected cells for analysis. On the other hand, we were able to isolate and analyze latent cells in a relatively short period of time (7 days). Multiple studies have shown that latently infected cells isolated from HIV-infected patients and NHP on long-term ART are enriched for expression of immune checkpoint molecules such as PD-1, CTLA-4, LAG-3 and TIGIT (17, 20, 21, 53). We did not find such enrichment in latently infected cells in our model. One possible explanation for this is that in our system cells are not reactivated in order to identify latently infected cells. Most of the studies that have identified PD-1 expression as a marker of latency are stimulating *in vitro* with a strong stimulus (e.g. PMA and Ionomycin) for up to 40 hours to induce HIV gene and protein expression which will have drastic effects on cell phenotype and function. An alternative explanation is that the short-term nature of our study is not capturing these observed effects of latent HIV infection.

Our model consists of tonsil CD4^+^ T cells which are more efficiently infected *ex vivo* by HIV strains than those from primary human peripheral blood lymphocytes (54) and we reasoned that they would allow for a larger pool of infected cells for down-stream analysis. Latent cells are often defined as ‘quiescent’ suggesting that the absence of cellular activity is a requirement. However, cellular activation markers are expressed at higher levels on T cells residing in tissues compared to circulating cells (55, 56) and multiple studies have shown a de-coupling between activation marker expression and productive HIV infection (57, 58), which was confirmed by our results as well. This suggests that surveillance of classical activation markers may not be sufficient to comprehend the cellular activation status as it relates to HIV transcriptional activity in the context of reactivation.

Given the difficulty of studying extremely rare and latent HIV-infected cell populations, especially in the context of viral suppression on ART, single cell analyses have allowed for enhanced resolution of *in vitro* and *ex vivo* studies (25, 30, 59). While frequencies of infected cells were low using the HIV_GKO_ system for this study, it was more robust than using patient-derived cells (1/100 *vs.* 1/million). We applied a plate-based approach to combine single cell sorting, flow cytometric phenotyping, and host and HIV gene transcript analyses to strictly define latently infected cells. Differential gene expression analysis revealed that latent cells expressed higher levels of the histone genes (*HIST1H1D, HIST1H4C*) confirming that chromatin modification is playing a role in latency establishment as was shown previously using this model (34). Histone modification controls transcription for host genes and integrated HIV (45, 60). The histone-related genes were significantly co-expressed on a single cell level with tubulin genes, mitochondrial-encoded cytochrome oxidase genes, and actin genes suggesting a connection between latency establishment and chemotaxis as reported previously in an alternative primary cell model of latency (28). Overall, these findings support the use of single cell analysis in studying HIV latency especially using technologies with advanced throughput such as 10X and Drop-seq. Identification of gene pathways in the HIV_GKO_ model that have been reported in the literature such as Sirtuin signaling (61) and upregulation of survival genes (25, 35) in latent infection reinforces the utility of this model to study the biology of and potential disruption of latent HIV infection.

We found *IL32* gene expression to be enriched in latent cells, but this transcript did not show co-expression with the histone-related genes suggesting it may be an additional molecular pathway involved in latency establishment. *IL32* is a cytokine expressed by T cells and to a lower extent in B cells and monocytes (62). The multiple isoforms of IL-32 have divergent properties and expression profiles whereby IL-32β is the most predominant and exhibits anti-inflammatory properties (along with IL-32α) and IL-32*γ* is pro-inflammatory (62). Compared to HIV-negative controls, all IL-32 isoforms are increased in plasma from HIV-infected individuals (62, 63). *Ex vivo* addition of IL-32 to CD4^+^ T cells from virally suppressed HIV^+^ individuals has shown conflicting results with regards to induction or suppression of HIV replication which may depend in part on the isoform used (62, 64, 65). Our results demonstrated an increase of *IL32* transcript in latent cells compared to uninfected or productively infected cells support a mechanism of transcriptional suppression of HIV as a way to establish and maintain latent infection and thus point to IL-32 as a potential target for latency reactivation.

The proposed mechanism of JAK1/2 inhibition on HIV replication is due to its effects on lowering cellular activation and increasing the pro-apoptotic protein Bcl-2, thereby reducing the lifespan of the reservoir-harboring cell while simultaneously blocking HIV-induced activation, which promotes HIV persistence and reservoir reseeding (40, 66–68). Collectively due to these tandem mechanisms, it is not surprising that baricitinib and ruxolitinib confer blockade of key events driving reservoir establishment and maintenance in this system. HIV_GKO_ is a single-round, replication deficient HIV, therefore, we cannot evaluate virus spreading in this model though it has been shown previously that ruxolitinib inhibits viral spreading *in vitro* with wild-type HIV infected cells (40), and has demonstrated efficacy towards blocking of HIV persistence markers and reservoir lifespan marker Bcl-2 in the A5336 study (39). Baricitinib (Olumiant.com) is cleared through the kidneys, significantly reducing potential for drug-drug interactions with co-administered agents that are cleared in the liver, which is a property of ruxolitinib (Jakafi.com). Further, baricitinib demonstrates a more favorable pharmacokinetic profile and efficacy in humans, at 2-4 mg once per day dosing for baricitinib *versus* 10-25 mg twice a day dosing for ruxolitinib. Recent reports have also shown that baricitinib can block key events associated with reservoir seeding and persistence in a murine model of HIV and across primary *in vitro* systems, further validating that baricitinib confers anti-HIV effects across systems within physiologically relevant concentrations found in humans (69).

Baricitinib has been shown able to block type 1 IFN-induced signaling (PMID: 30002661) in a concentration dependent manner. We also know from human studies in the COVID setting that baricitinib blocks virus induced IFN signaling in hepatocytes which correlates well with favorable clinical outcomes (PMID: 33187978). While there is nothing in the literature yet about baricitinib in the HIV space, these findings suggest that JAK1/2 inhibition by baricitinib may also block HIV-induced IFN and lead to reduction in reservoir. As baricitinib represents a next-in class JAK1/2 inhibitor with an improved safety and efficacy profile *versus* ruxolitinib, it is also promising that our data demonstrate an increased efficacy profile compared to ruxolitnib. Together, the data reported provide a key foundation towards establishing that JAK1/2 inhibition, and in particular baricitinib, represent a potential modality towards reservoir reduction in key cells. Further, our findings demonstrate for the first time that physiologically relevant concentrations of baricitinib can reduce the HIV reservoir in lymphoid tissue derived cells. These data coupled with the body of work with this class of agents including humans (A5336), will provide an informed, robust, and mechanistic framework from which to build additional human studies with baricitinib for the indication of HIV cure.

## Data Availability Statement

The raw data supporting the conclusions of this article will be made available by the authors, without undue reservation.

## Ethics Statement

The studies involving human participants were reviewed and approved by Institutional Review Boards of University of Miami (IRB# 20140200/CR00004416). Written informed consent to participate in this study was provided by the participants’ legal guardian/next of kin.

## Author Contributions

LA, CG, SuP, SR, RP, SW, RS, and SaP provided intellectual input and contributed to the experimental design. LA and SR performed data collection. CG, RS, EB, EV, and RY provided critical reagents and/or tissue. LA, CG, and LP performed data analysis and interpretation. LA and CG wrote the manuscript. All authors provided critical feedback to produce the final manuscript. All authors contributed to the article and approved the submitted version.

## Funding

This work was supported by grants awarded to LA and SW from the Institute of AIDS and Emerging Infectious Diseases and the Miami Center for AIDS Research (CFAR) at the University of Miami Miller School of Medicine funded by a grant (P30AI073961) from the National Institutes of Health (NIH), which is supported by the following NIH Co-Funding and Participating Institutes and Centers: NIAID, NCI, NICHD, NHLBI, NIDA, NIMH, NIA, NIDDK, NIGMS, FIC AND OAR. RS and CG were supported by NIH grant RO1-MH-116695 and in part by Emory University’s CFAR NIH grant P30-AI-050409.

## Conflict of Interest

The authors declare that the research was conducted in the absence of any commercial or financial relationships that could be construed as a potential conflict of interest.

## Publisher’s Note

All claims expressed in this article are solely those of the authors and do not necessarily represent those of their affiliated organizations, or those of the publisher, the editors and the reviewers. Any product that may be evaluated in this article, or claim that may be made by its manufacturer, is not guaranteed or endorsed by the publisher.
