# Supplementary material for: The Effect of JAK1/2 Inhibitors on HIV Reservoir Using Primary Lymphoid Cell Model of HIV Latency
Source: Front Immunol. 2021 Aug 31;12:720697. doi: 10.3389/fimmu.2021.720697 (PMC8438319; doi:10.3389/fimmu.2021.720697)
Supplement: Supplementary file 3 [file Image_1.pdf]

Supplemental Figure 1

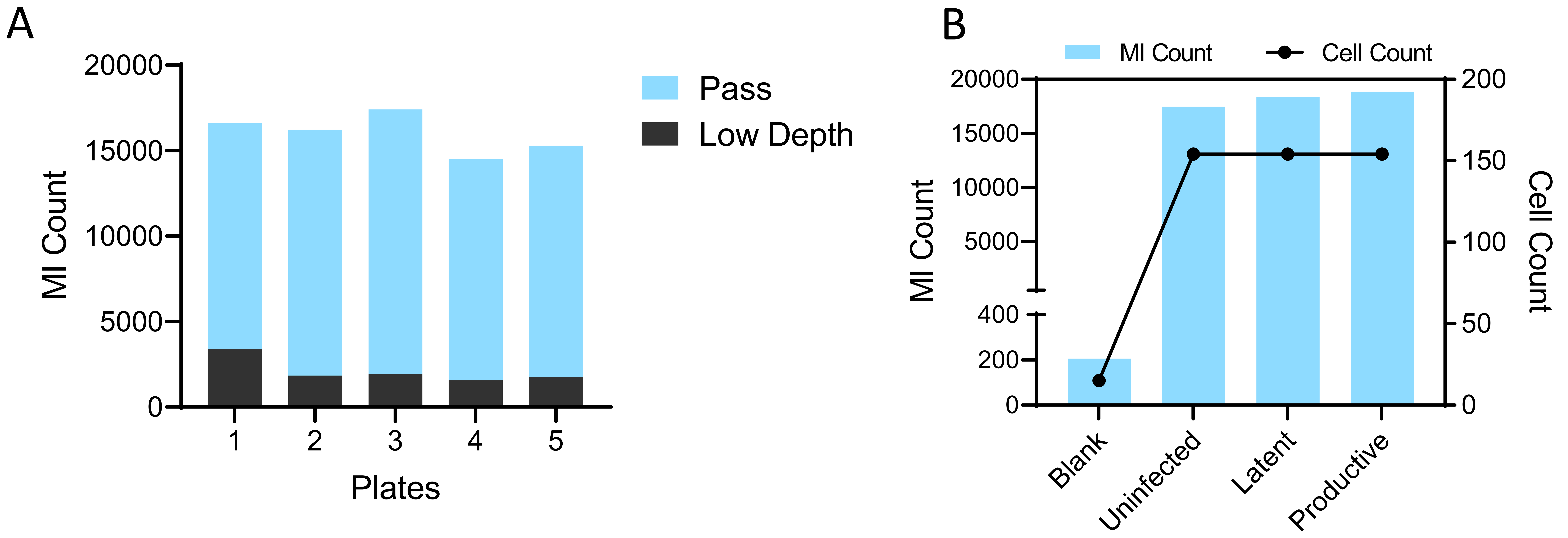

Supplemental Figure 1. Single Cell RNA Seq in HIV_{GKO} model of latency. A) Molecular Index (MI) counts across all 5 plates collected from a single donor and 3 independent experiments. B) MI counts and number of cells sorted per infection population and blank control wells in which no cells were sorted.

Index Sorting Data:

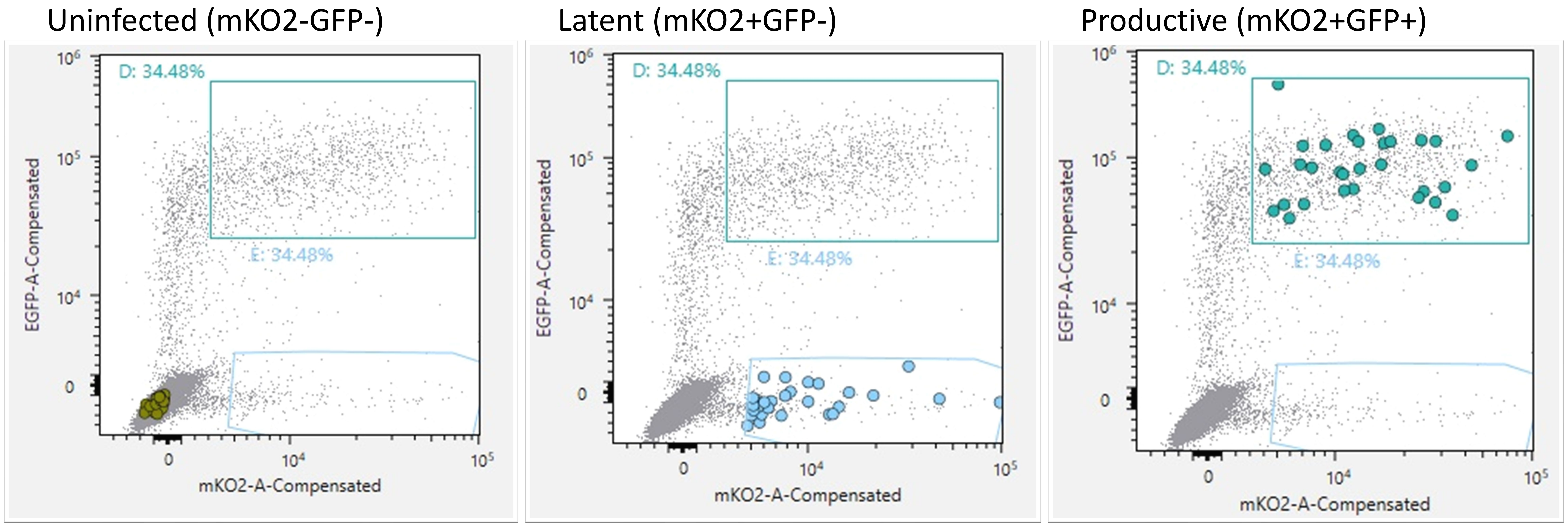

Supplemental Figure 2. Dot Plots showing fluorescent profiles for GFP and mKO2 expression of single cells sorted using Sony SH800 for each sorted population as indicated. Gray dots indicate total LIVE cells acquired and large colored dots represent sorted cells.

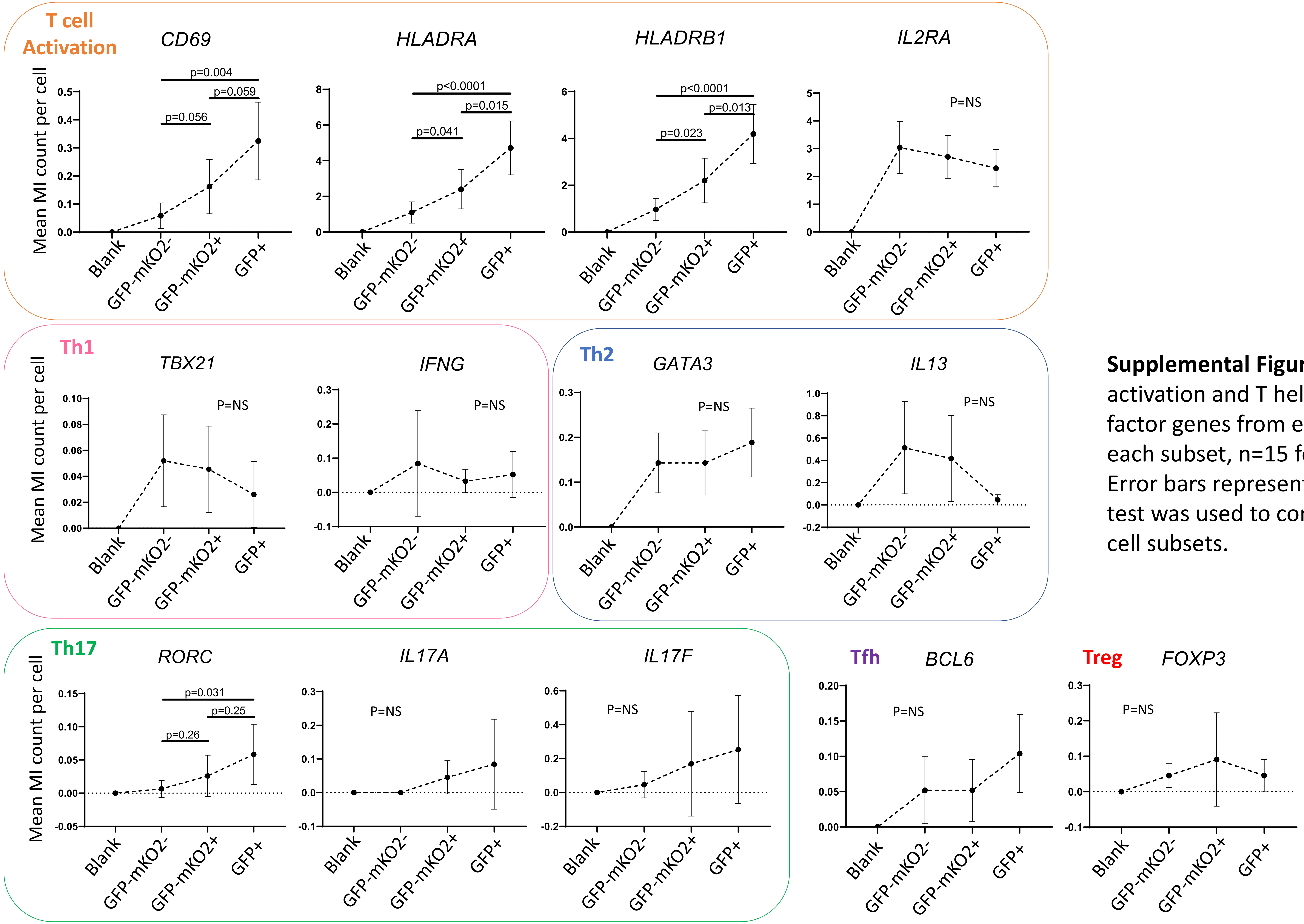

Supplemental Figure 3. Mean MI count per cell for T cell activation and T helper subset cytokine/transcription factor genes from each sorted cell subset (n=154 cells each subset, n=15 for Blank wells containing no cell). Error bars represent 95% Confidence interval. Unpaired t test was used to compare means between the different cell subsets.

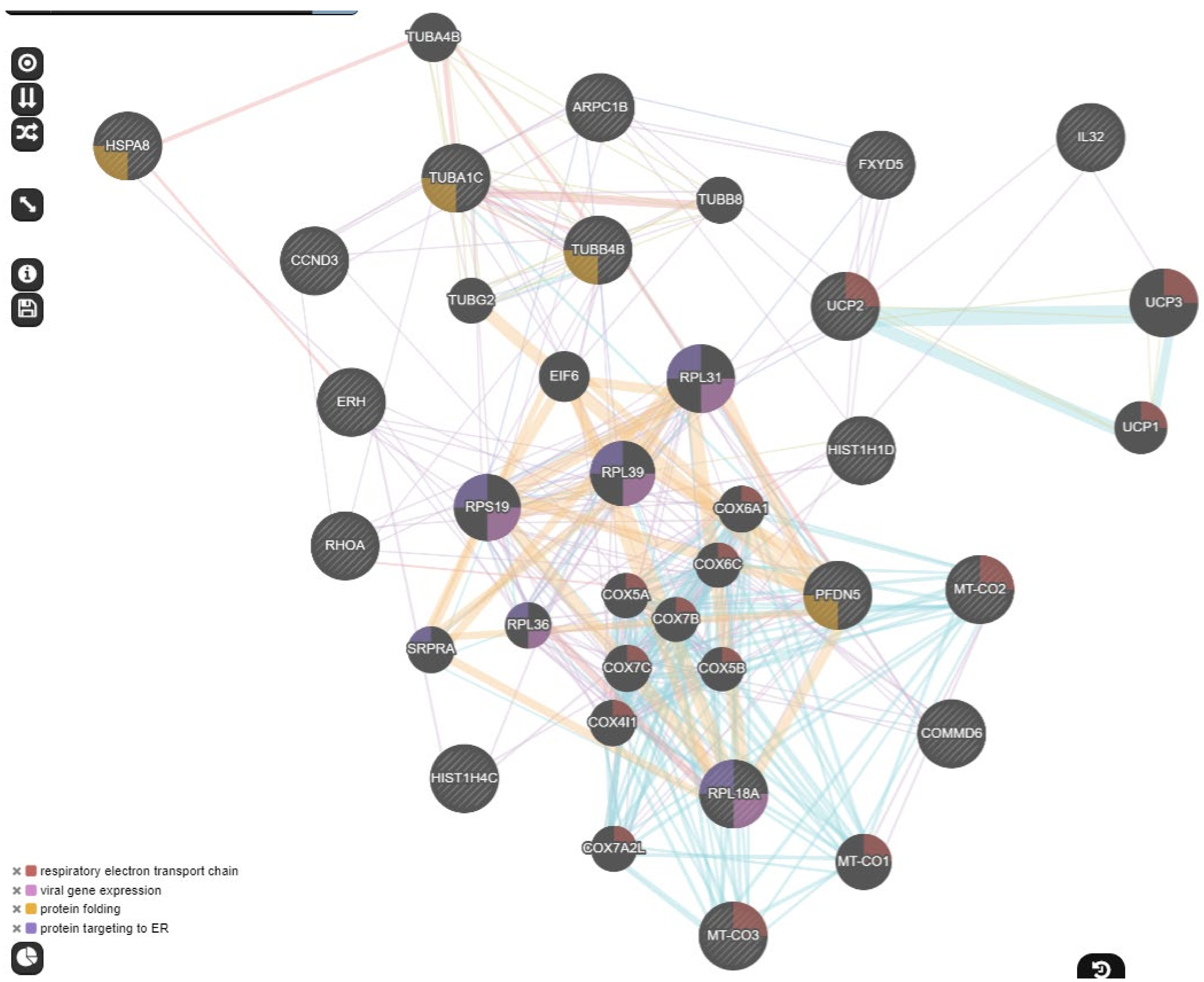

Supplemental Figure 4. Network analysis for genes differentially expressed in latent and uninfected cells using GENEMANIA.org. Larger circles denote genes input for network analysis. Colored wedges indicate specific functions attributed to the gene as indicated by Function Legend in bottom-left corner.

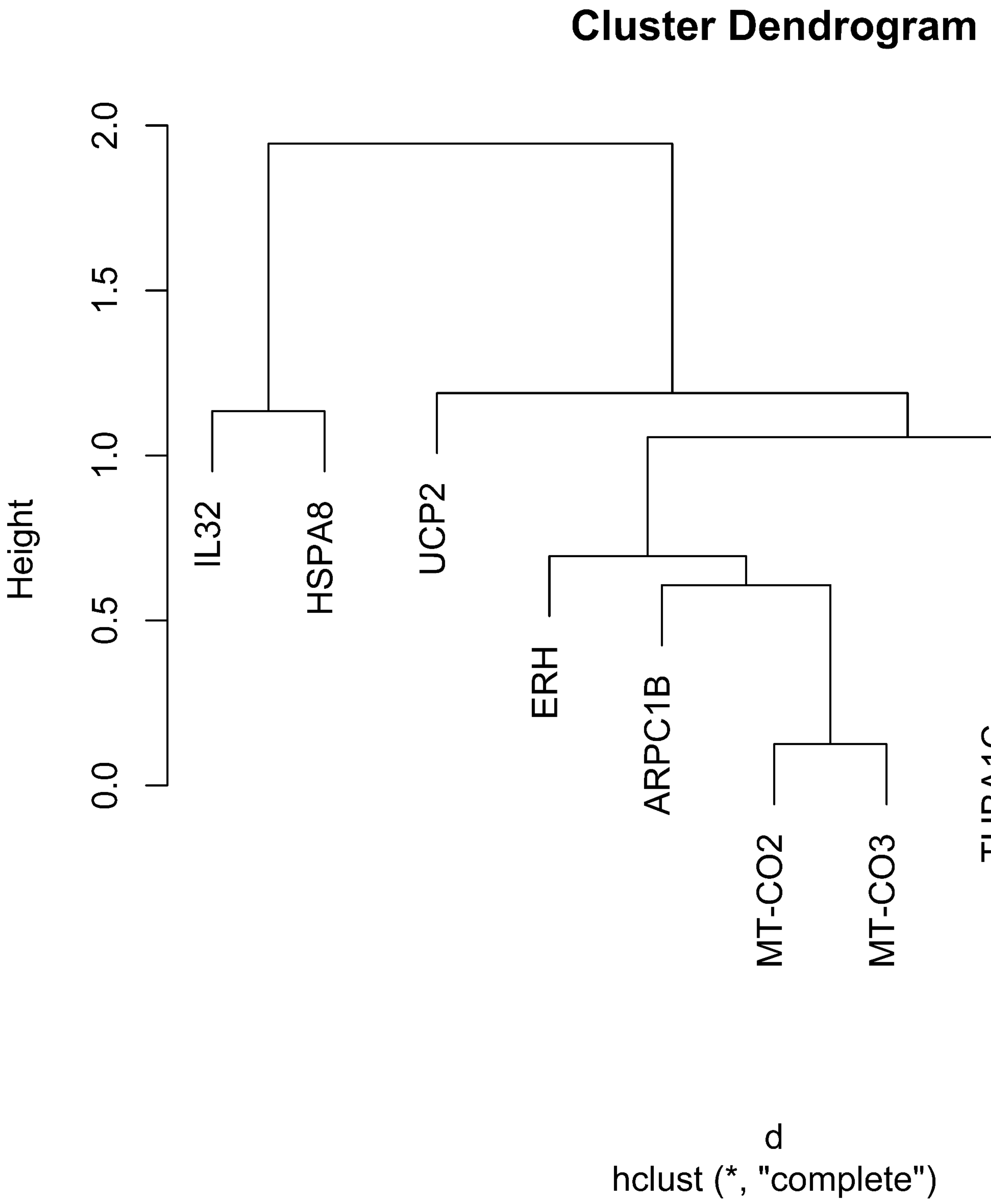

Supplemental Figure 5. Phylogenetic tree showing clustering for co-expression of latent signature gene set on the single cell level using all cells analyzed.
