# Supplementary material for: The Effect of JAK1/2 Inhibitors on HIV Reservoir Using Primary Lymphoid Cell Model of HIV Latency
Source: Front Immunol. 2021 Aug 31;12:720697. doi: 10.3389/fimmu.2021.720697 (PMC8438319; doi:10.3389/fimmu.2021.720697)
Supplement: Supplementary file 4 [file Table_1.docx]

**Supplementary Table 1. Details for Commercially Available Monoclonal Antibodies used in Flow**

| **Antibody** | **Clone** | **Company + Catalog Number** |
| --- | --- | --- |
| CD4 PerCp-Cy5.5 | L200 | BD 552838 |
| CD45RO BV650 | UCHL1 | BD 563750 |
| CD69 AlexaFluor 700 | FN50 | Biolegend 310922 |
| HLA-DR APC-Cy7 | L243 | Biolegend 307617 |
| CD25 BV605 | 2A3 | BD 562660 |
| PD-1 PE-Cy7 | EHI2.1 | BD 561272 |
| CXCR5 AlexaFluor 647 | RF8B2 | BD 558113 |

**Cytometry Assays.**

**Supplementary Table 2. Related to Figure 2A and B, CD45RO expression on Total, Productive and Latent infected CD4+ T cells.**

| **Tonsil Donor** | **Total (CD45RO+ %)** | **Productive (CD45RO+ %)** | **Latent (CD45RO+ %)** |
| --- | --- | --- | --- |
| 1050 | 89.5 | 97.5 | 99.0 |
| 1051 | 90.1 | 95.1 | 100.0 |
| 1052 | 62.0 | 93.3 | 94.5 |
